# Influence of Multiple Binding Sites on the Supramolecular Assembly of *N*-[(3-pyridinylamino) Thioxomethyl] Carbamates

**DOI:** 10.3390/molecules27123685

**Published:** 2022-06-08

**Authors:** Kelly N. Shunje, Boris B. Averkiev, Christer B. Aakeröy

**Affiliations:** Department of Chemistry, Kansas State University, Manhattan, KS 66506, USA; knshunje@ksu.edu (K.N.S.); averkiev@ksu.edu (B.B.A.)

**Keywords:** hydrogen bonds, halogen bonds, binding preference, intermolecular interactions

## Abstract

In this study, we investigated how the presence of multiple intermolecular interaction sites influences the heteromeric supramolecular assembly of *N*-[(3-pyridinylamino) thioxomethyl] carbamates with fluoroiodobenzenes. Three targets—R-*N*-[(3-pyridinylamino) thioxomethyl] carbamate (R = methyl, ethyl, and isobutyl)—were selected and crystallized, resulting in three parent structures, five co-crystals, and one co-crystal solvate. Three hydrogen-bonded parent crystal structures were stabilized by N-H···N hydrogen bonding and assembled into layers that stacked on top of one another. Molecular electrostatic potential surfaces were employed to rank binding sites (Npyr > C=S > C=O) in order to predict the dominant interactions. The N-H⋯H hydrogen bond was replaced by I⋯Npyr in 3/6 cases, I⋯C=S in 4/6 cases, and I⋯O=C in 1 case. Interestingly, the I⋯C=S halogen bond coexisted twice with I⋯Npyr and I⋯O=C. Overall, the MEPs were fairly reliable for predicting co-crystallization outcomes; however, it is crucial to also consider factors such as molecular flexibility. Finally, halogen-bond donors are capable of competing for acceptor sites, even in the presence of strong hydrogen-bond donors.

## 1. Introduction

The ability to foresee and control the outcome of the organization and assembly of molecules is a highly coveted goal in the bottom-up synthesis of functional materials. Achieving this requires a better understanding of non-covalent intermolecular interactions between the building blocks, and of the overall crystal packing arrangement of the molecules. From a practical perspective, manipulation of intermolecular interactions has been used to establish structure–property–function correlations in applications such as molecular recognition [1], the design of mechanically flexible molecular crystals [2,3,4,5,6], controlling thermal expansion behavior [7], and the design of molecular capsules [8]. In addition, an improved understanding of intermolecular interactions is essential if we want to control key crystallization events that lead to synthon polymorphism [9,10,11].

Hydrogen bonding is the most extensively studied non-covalent interaction, followed by halogen bonding [12,13]. Both display comparable strength and directionality, which can make it difficult to predict outcomes of how they will affect supramolecular assembly when functional groups that can accept hydrogen bonds and halogen bonds equally well are present in a system. Several strategies have been proposed to predict the outcomes in cases where multiple intermolecular interaction sites are present, such as hydrogen-bond energy (HBE) and hydrogen-bond propensity (HBP) [14,15]. Computing molecular electrostatic potential (MEP) maps is another method for analyzing the distribution of electron densities in molecular systems where the highest positive potential is classified as the best donor, and the highest negative potential as the best acceptor [16]. The ranking of these donor/acceptor sites can also shed light on which sites will preferentially bind to one another.

To establish binding preference patterns in systems where both interactions can coexist, Shimazu et al. carried out co-crystallization experiments of dipyridyloxalamide ligands with tetrafluorodiiodobenzenes [17]. The competing acceptors as ranked by MEP were pyridyl nitrogen and the carbonyl oxygen of the oxalamide groups, and neither displayed specific binding preference to the tetrafluorodiiodobenzenes over the other. Resnati et al. also demonstrated that halogen and hydrogen bonding can exist orthogonally to construct porous organic frameworks [18].

**Scheme 1:**
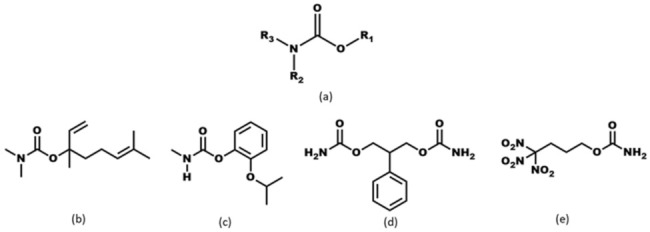
(**a**) Carbamate moiety and carbamate-bearing molecules, (**b**) pepperwood, (**c**) Baygon (**d**) felbamate (**e**) trinitrobutyl nitrocarbamate.

Of continued interest in our group are efforts to establish binding preferences for systems with multiple acceptors that have significantly different electrostatic strengths. In previous studies, we carried out binding preference studies—including on heteroaryl-2-imidazoles with one hydrogen-bond (HB) donor and two or three different acceptor sites (ranked by MEPs)—using monotopic halogen-bond (XB) donor co-formers [19,20,21,22]. It was observed that XB donors outperformed the HB donor for the most electrostatically attractive acceptor site. In the present study, we explore a new target library decorated with the carbamate functional group. Carbamate-bearing molecules and their derivatives [23,24,25,26,27] (Scheme 1) have wide-ranging applications in areas such as pesticides [28,29,30,31], active pharmaceutical ingredients [32,33], and energetic materials [34,35,36]. Carbamates are also versatile compounds for crystal engineering because they contain multiple acceptor and donor sites, such as carbonyl oxygen atoms, pyridyl nitrogen atoms, and C=S. However, this versatility also makes them challenging from an a priori design perspective.

**Scheme 2:**
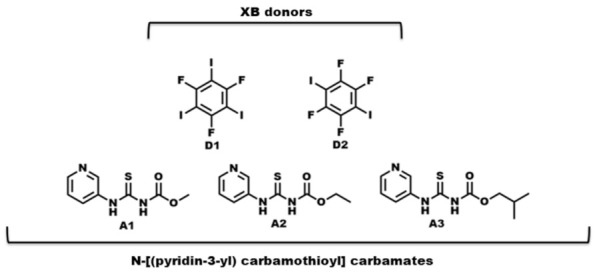
Molecular structures of R-*N*-[(3-pyridinylamino) thioxomethyl] carbamates and halogen-bond donors used in this study.

In this study, we address five specific questions:

◾ What is the impact of varying chain length (R = methyl, ethyl, and isobutyl) on the crystal structures of the target molecules **A1**–**A3** (Scheme 2)?
◾ Which structure-directing synthons are formed when targets are co-crystallized [37,38,39] with halogen-bond donor co-formers?
◾ What binding preference is observed in the solid state when hydrogen-bond and halogen-bond donors (**D1** and **D2**) compete for three different acceptor sites (Scheme 2)?
◾ Does the supramolecular assembly change for different targets if we introduce the same XB donors as co-formers for co-crystallization?
◾ How reliable are MEP rankings/predictions when multiple acceptors are present on the target molecules?

**Scheme 3:**
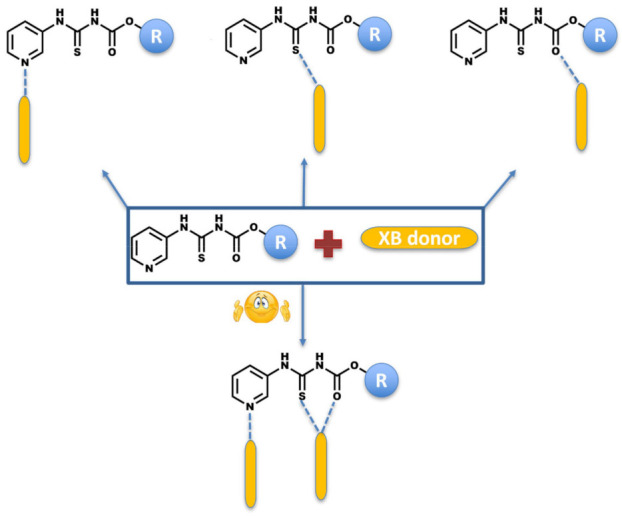
Postulated primary intermolecular interactions in co-crystals of target molecules and halogen-bond donors.

An attempted co-crystallization of the target compounds with XB donors can result in the target and the co-former precipitating separately, in which case it would be a recrystallization. On the other, if halogen bonding is structure-directing, it can lead to the formation of a co-crystal via various postulated synthons (Scheme 3).

## 2. Results

**Figure 1:**
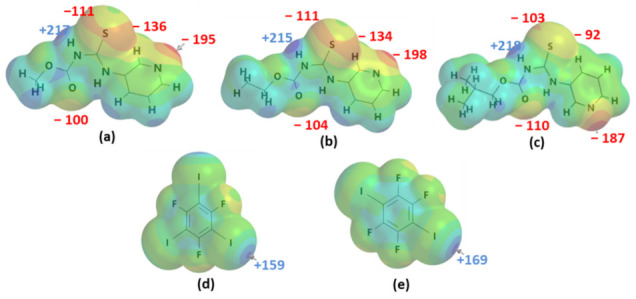
Molecular electrostatic potentials (kJ/mol) of the targets (**a**–**c**) **A1**–**A3**, respectively, and (**d**,**e**) XB donors **D1**–**D2,** respectively, showing electron-rich (red) and electron-deficient (blue) regions.

Molecular electrostatic potentials were used for ranking the donor and acceptor ability of hydrogen-bond donors, halogen-bond donors, and acceptor sites by considering only the electrostatic component. The R-*N*-[(3-pyridinylamino) thioxomethyl] carbamates’ targets contain acceptors such as carbonyl oxygen atoms (C=O), pyridyl nitrogen atoms (Npyr), and C=S, as well as the hydrogen-bond donor N-H (Figure 1a–c). The sulfur atom of the C=S group has two different regions of electron density, as shown below. The two regions have significantly different potentials (≤23 kJ/mol) for **A1** and **A2**, while a difference of ≤ 10 kJ/mol is calculated for **A3**. The halogen-bond donors range from 159 to 169 kJ/mol (Figure 1d,e). The target molecules have multiple donating (215 to 218 kJ/mol) and accepting sites (−92 to −198 kJ/mol), and the acceptors can be ranked in the order Npyr > C=S > C=O. The question, then, is where will binding occur if we introduce XB donors as co-formers for co-crystallization?

**Figure 2:**
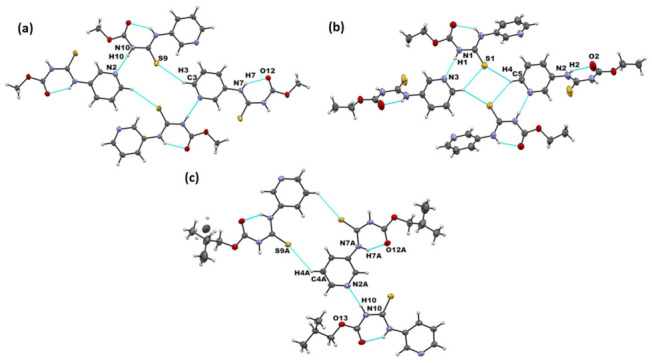
Primary non-covalent interactions (in blue) in the crystal structures of (**a**) **A1**, (**b**) **A2**, and (**c**) **A3**.

We obtained crystals suitable for SCXRD analysis of **A1** and **A3**. The crystal structure of **A2** has previously been reported [40]. In the crystal structures of **A1**–**A3**, the primary intermolecular interactions were N–H⋯Npyr complemented by an intramolecular N–H⋯O hydrogen bond. In addition, all three structures formed C-H···S=C interactions (Figure 2a–c). Overall, connectivity in the crystal structures of the parent molecules showed the following preferred mode of interaction: N-H···Npyr as the primary interaction, generating chain-like assembly and C-H···S=C dimer formation in all three targets. This matches with the expected MEP-based interaction hierarchy, which assumes that the best donor preferentially binds to the best acceptor.

**Figure 3:**
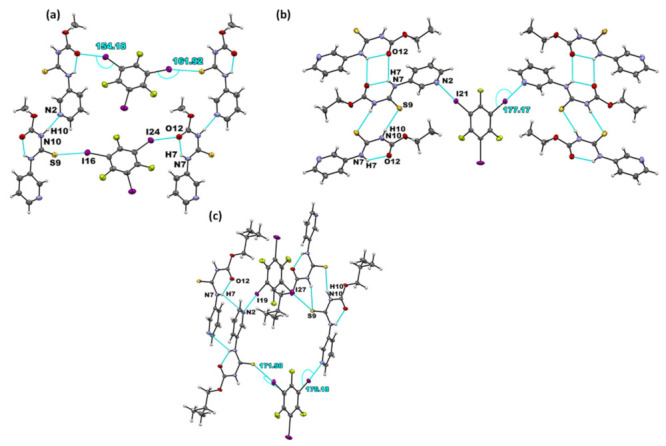
Primary non-covalent interactions (in blue) in the crystal structures of (**a**) **A1·D1**, (**b**) **(A2)_2_·D1**, and (**c**) **A3·D1**.

Six co-crystals of each target compound with 1,3,5-trifluoro-2,4,6-triiodobenzene (**D1**) and 1,2,4,5-tetrafluoro-3,6-diiodobenzene (**D2**) were synthesized: **A1·D1**, **(A2)_2_·D1**, **A3·D1**, **(A1)_2_·D2**, **A2·D2**, and **(A3)_2_·D2**. In **A1·D1,** the hydrogen-bonded chains observed in parent molecules between the adjacent **A1** molecules via N–H⋯Npyr interactions are retained. In addition, the **D1** donor acts as a crosslink between **A1** molecules through I⋯S=C and I⋯O=C halogen bonds (Figure 3a). In **(A2)_2_·D1**, the binding preference is through I⋯Npyr halogen bonds, while the parent molecules form dimers through N-H···S=C and N-H···O=C interactions (Figure 3b). It is worth noting that the N–H⋯Npyr hydrogen bond in **A2** is replaced by the new I⋯Npyr halogen bond. Co-crystallization of **A3** and **D1** also yielded co-crystals via I⋯Npyr, and unlike in **(A2)_2_·D1** where I⋯Npyr was the only halogen-bond, an I⋯S=C halogen bond was also observed. In addition, the parent molecules interacted by forming (N-H···S=C) dimers. The remaining acceptor, C=O on **A3**, did not participate in any intermolecular bonds of note.

**Figure 4:**
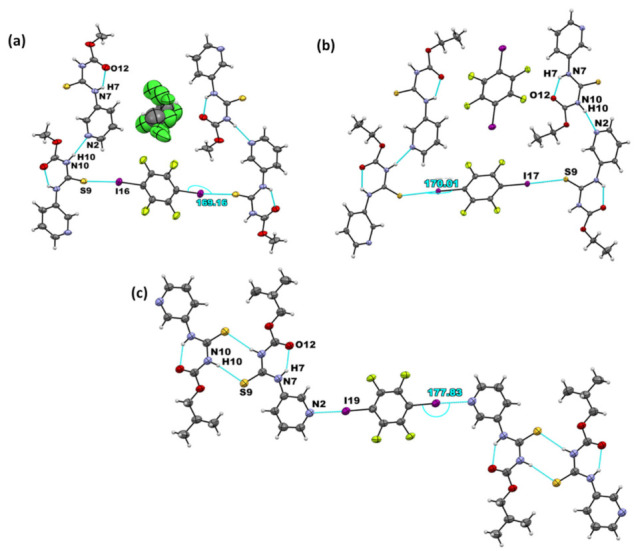
Primary non-covalent interactions (in blue) in the crystal structures of (**a**) **(A1)_2_·D2**, (**b**) **A2·D2,** and (**c**) **(A3)_2_·D2**.

The combination of **D2** with the R-N-[(3-pyridinylamino) thioxomethyl] carbamates resulted in the formation of three co-crystals. Furthermore, **(A1)_2_·D2** appeared as a solvate with a disordered chloroform in the asymmetric unit. Similar to **A1·D1**, a hydrogen-bonded chain was present between adjacent target molecules via N–H⋯Npyr interactions. In this co-crystal, the donor exclusively binds to the C=S acceptor, providing a bridge between two target molecules through I···S=C (Figure 4a). The same primary synthons and assemblies are present in the structure of **A2·D2,** as shown in Figure 4b. **(A3)_2_·D2**, on the other hand, yielded halogen-bonded co-crystals via I⋯Npyr, while the parent molecules formed a dimer through N-H···S=C (Figure 4c).

## 3. Discussion

**Figure 5:**
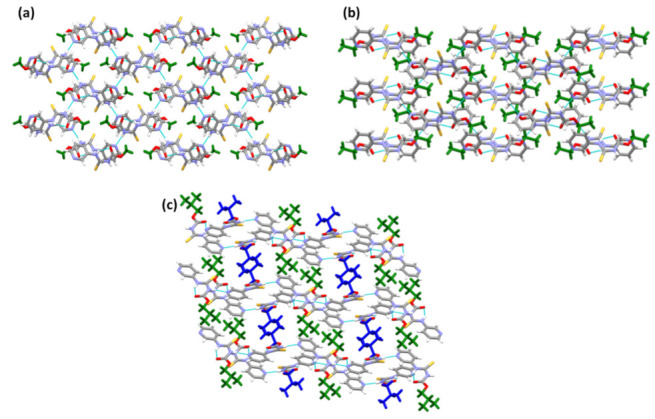
Crystal packing in the structures of (**a**) **A1**, (**b**) **A2**, and (**c**) **A*3***.

All three crystal structures of the target compounds themselves showed identical hydrogen-bond motifs. A packing analysis was carried out to investigate the effect that aliphatic chains with varying lengths (R= methyl, ethyl, and isobutyl) may have on synthon robustness. The crystal structures of **A1** and **A2** contain layers that stack on top of one another, with no notable interlayer interactions. Furthermore, the alkyl groups (in green) point towards one another, as seen in the packing units of **A1** and **A2** (Figure 5a,b). For **A3**, similar to the methyl and ethyl groups, the isobutyl groups point toward one another (Figure 5c). However, because there are two symmetry-independent molecules present in the asymmetric unit, the tails can be differentiated (indicated by blue and green coloring in the figure). Moreover, because of the bulkier nature of the isobutyl group, the molecules aggregate into hydrophobic layers of the alkyl chain and pi-stacked layers of the aromatic rings. The packing index was calculated using PLATON [41], and was used to characterize the total efficiency and compactness of the packing in the targets. The packing index was found to be 70.3% for **A1** and 66.4% for **A2** and **A3**. Overall, the packing was similar in all three target structures despite the increase in the alkyl chain length, but it is notable that the packing efficiency was slightly reduced in the structures of the compounds containing ethyl and isobutyl groups.

**Figure 6:**
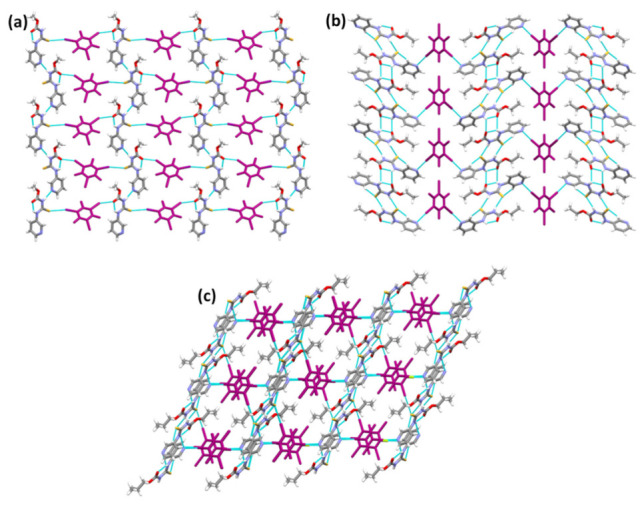
Crystal packing in the structures of (**a**) **A1·D1**, (**b**) **(A2)_2_·D1**, and (**c**) **A3·D1**.

In **A1·D1** and **A3·D1**, the target molecules assemble into chains, and **D1** provides a crosslink between adjacent chains (Figure 6a,c). Unlike **A1·D1** and **A3·D1**, in **(A2)_2_·D1,** the target molecules aggregate to form sheets connected by **D1** molecules (Figure 6b). Despite having different intermolecular interactions, the co-crystals adopt similar packing (Figure 6a–c).

**Figure 7:**
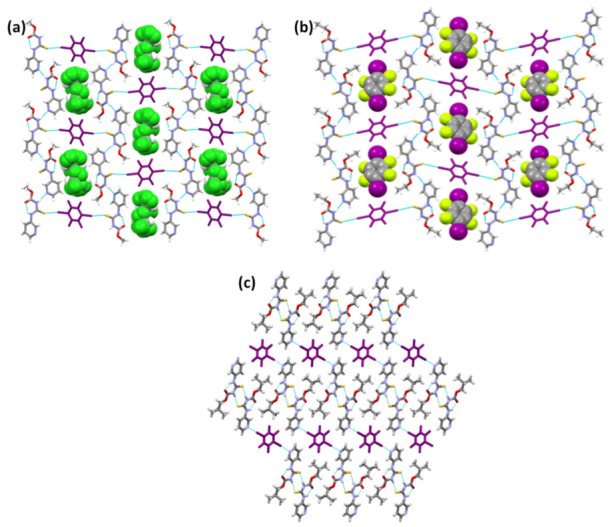
Crystal packing in the structures of (**a**) **(A1)_2_·D2**, (**b**) **A2·D2,** and (**c**) **(A3)_2_·D2.**

The chloroform and **D2** molecules in the lattice of **(A1)_2_·D2** and **A2·D2,** respectively (Figure 7a,b), are primarily space-filling molecules, and do not seem to engage in any strong interactions with neighboring molecules. Despite **(A1)_2_·D2** being a solvate and **A2·D2** being a pure co-crystal, both structures adopt the same packing. Furthermore, **(A1)_2_·D2** and **A2·D2** exhibit similar packing behavior to **A1·D1** and **A3·D1**, where target molecules form chains interconnected by the co-formers through halogen bonds. **(A3)_2_·D2** is different from **(A1)_2_·D2** and **A2·D2** in that **(A3)_2_·D2** forms I⋯Npyr halogen bonds with **D2**, while the latter both form I⋯S=C halogen bonds. Although binding preference to S=C is observed in **(A1)_2_·D2** and **A2·D2,** it is still not obvious why the binding preference switches to I⋯Npyr in **(A3)_2_·D2** (Figure 7c). Overall, the postulated interactions in Scheme 2 were observed either by themselves or as part of a series in the six co-crystals.

All crystal structures obtained were compared to the outcomes suggested by MEP calculations. In this study, the best donor and acceptor were predicted to be the N-H and Npyr of the target, respectively. If the best donor binds to the best acceptor, N–H⋯Npyr hydrogen bonds are retained, thus leaving the C=S and C=O acceptors to be free to form halogen bonds with the XB donors. However, if we assume that XB donors would preferentially bind to stronger acceptor sites, then the acceptors can be ranked as Npyr > C=S > C=O, based purely on electrostatics.

Structural competition was not expected between the acceptors Npyr and C=S, since their MEPs have about 60 kJ/mol difference. The I⋯Npyr halogen bond was present in 3/6 structures, while the I⋯S=C halogen bond was found in 4/6 cases. In one of the structures, both interactions were present, suggesting that MEPs underestimate the competing ability of the C=S moiety. Competition between C=S and C=O was expected to be moderate, since their difference in MEP was ∼30 kJ/mol. The I⋯S=C bond was observed in 4/6 structures, while I⋯O=C was observed only once. The MEPs proved to be effective for predicting the outcomes of the attempted co-crystallizations.

The % van der Waals reductions [42] were examined to get a semi-qualitative sense of the strengths of the halogen-bond interactions observed. Analysis of the % vdW reductions in Table 1 reveals that the I⋯Npyr bond was stronger (16–20%; 176.2–177.8°) compared to the I⋯C=S bond (11–14%; 161.9–171.9°) and I⋯O=C bond (154°). The experimental % vdW reductions were correlated with the MEP predictions. However, a careful analysis of the impact of molecular geometry could also be important for improved prediction accuracy, given that the parent molecules are flexible.

**Figure 8:**
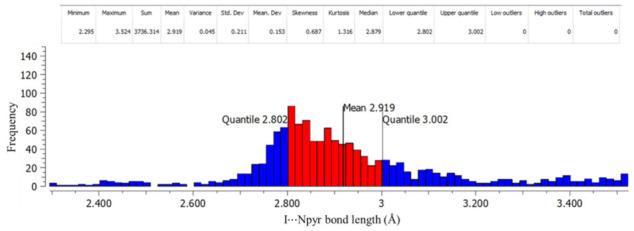
Histogram showing bond length against frequency for I⋯Npyr halogen bonds.

The Cambridge Structural Database (CSD)’s ConQuest version 2021.1.0 [43] was used to provide a larger context through a search for the following intermolecular interactions: I⋯Npyr, I⋯S=C, and I⋯O=C. Figure S4 shows a summary of the contact descriptors used for the CSD search. The search yielded 832 individual crystal structures containing an I⋯Npyr halogen bond, 110 crystal structures containing I⋯S=C, and 30 structures containing I⋯O=C. Figure 8 and Figures S5 and S6 show the histogram plots of frequency against bond length, along with descriptive statistical data for each type of interaction. The histograms and descriptive statistical data were plotted using Mercury version 2021.1.0 [44]. Of the 832 CSD hits containing the I⋯Npyr halogen bond, the interactions had 1280 total bond lengths. According to the histogram for I⋯Npyr below, 50% of the bond lengths were in the midrange (2.80–2.92 (Å)), with a mean of 2.92 Å, while 24.8% were in the lower range (2.30–2.80 (Å)) and 25.2% in the upper range (3.00–3.52 (Å)). The CSD results show that the I⋯Npyr bond was the most prevalent, and the bonds obtained in our crystal structures fit into the midrange, where the majority of the reported structures appear.

For the I⋯S=C bond, the 110 CSD hits gave a distribution of 184 various bond lengths. The histogram (Figure S5) shows that 50% of those bond lengths were in the midrange (3.18–3.65 (Å)), with a mean of 3.36 Å, while 25% were in the lower range (2.49–3.18 (Å)) and 25% in the upper range (3.65–3.78 (Å)). Again, the I⋯S=C bond lengths obtained in our crystal structures were similar to the majority of the reported data. For the I⋯O=C bonds, we obtained only 30 hits (34 bond lengths), which is significantly fewer hits than for I⋯Npyr and I⋯S=C. This suggests that the interaction is not as prevalent as was the case in our crystal structures, appearing only once. Overall, the histogram (Figure S6) shows that the bond lengths are distributed with a mean of 3.18 Å, and 50% of the bond lengths are in the range 3.07–3.28 Å. It is worth noting that despite fewer CSD hits for I⋯O=C bonds, in our case it was not surprising that this interaction was less prevalent, since O=C formed an intramolecular (N-H⋯O=C) hydrogen bond in all structures.

## 4. Materials and Methods

### 4.1. Reagents and General Methods

All reagents were used as received without any further purification. MEPs were calculated using density functional theory on molecules optimized in the gas phase using the Spartan 08 program at the B3LYP/6-311++G** level of theory [45]. All of the NMRs were recorded on a Bruker 400 MHz spectrophotometer. Single-crystal X-ray diffraction (SCXRD) data were obtained using a Rigaku XtaLAB Synergy-S with a CuKα source. The melting points were collected using a TA Instruments DSC Q20 differential scanning calorimeter. Targets were synthesized using modified versions of reported procedures [46,47].

### 4.2. Synthesis of Methyl-N-[(3-pyridinylamino) Thioxomethyl] Carbamate (**A1**)

Methyl chloroformate (0.78 mL, 10 mmol) was added dropwise to a solution of ethyl acetate containing potassium thiocyanate (1.17 g, 12 mmol) [47]. The reaction mixture was heated at 75 °C for 3 h. KCl was filtered off, and 3-aminopyridine (0.94 g, 10 mmol) was added to the filtrate. The reaction mixture was heated under reflux for a further 5 h. The mixture was then cooled to room temperature, followed by vacuum filtration. After evaporating the solvent in a vacuum, the product was obtained by washing the precipitate three times with 75% ethanol. The product was a white solid with a yield of 68%, m.p: 172–175 °C. ^1^H NMR (400 MHz, chloroform-d) δ 11.50 (s, 1H), 8.70 (d, J = 2.6 Hz, 1H), 8.51 (dd, J = 4.9, 1.5 Hz, 1H), 8.39 (s, 1H), 8.25 (ddd, J = 8.3, 2.8, 1.5 Hz, 1H), 7.36 (dd, J = 8.3, 4.8 Hz, 1H), 3.88 (s, 3H). ^13^C NMR (101 MHz, CDCl_3_) δ 178.57, 153.31, 147.68, 145.60, 134.52, 131.73, 123.29, 53.74.

### 4.3. Synthesis of Ethyl-N-[(3-pyridinylamino) Thioxomethyl] Carbamate (**A2**)

Ethyl chloroformate (0.96 mL, 10 mmol) and ethyl acetate were mixed in a round-bottomed flask [40,46]. To this solution, potassium thiocyanate (1.17 g, 12 mmol) and N,N,N′,N′-tetramethylethylenediamine (TMEDA) (0.02 mL, 0.1 mmol) were added, and the reaction mixture was stirred at room temperature for 5 h. 3-Aminopyridine (0.94 g, 10 mmol) was then slowly added to the reaction mixture under constant stirring. The reaction mixture was stirred at room temperature for another 5 h. The progress of the reaction was monitored using TLC. Upon completion, the solvent was evaporated under vacuum, and the product was obtained by washing the precipitate three times each with 10 mL of 75% ethanol and 15 mL of water. The product was a white solid with a yield of 75%, m.p: 164–165 °C. ^1^H NMR (400 MHz, chloroform-d) δ 11.56 (s, 1H), 8.71 (d, J = 2.6 Hz, 1H), 8.60–8.56 (m, 1H), 8.52 (dd, J = 4.8, 1.5 Hz, 1H), 8.26 (ddd, J = 8.4, 2.8, 1.6 Hz, 1H), 7.39–7.31 (m, 1H), 4.31 (q, J = 7.1 Hz, 2H), 1.37 (t, J = 7.1 Hz, 3H).^13^C NMR (101 MHz, CDCl_3_) δ 178.81, 152.98, 147.59, 145.60, 134.59, 131.72, 123.26, 63.31, 14.21.

### 4.4. Synthesis of Isobutyl-N-[(3-pyridinylamino) Thioxomethyl] Carbamate (**A3**)

Isobutyl chloroformate (1.30 mL, 10 mmol) and ethyl acetate were mixed in a round-bottomed flask. To this solution, potassium thiocyanate (1.17 g, 12 mmol) and TMEDA (0.02 mL, 0.1 mmol) were added, and the reaction mixture was stirred at room temperature for 5 h. 3-Aminopyridine (0.94 g, 10 mmol) was then slowly added to the reaction mixture under constant stirring. The reaction mixture was stirred at room temperature for another 5 h. After evaporating the solvent in a vacuum, the product was obtained by washing the precipitate with 75% ethanol and water. The product was a white solid with a yield of 71%. m.p: 123–125 °C. ^1^H NMR (400 MHz, chloroform-d) δ 11.56 (s, 1H), 8.71 (d, J = 2.6 Hz, 1H), 8.65 (s, 1H), 8.51 (d, J = 4.8 Hz, 1H), 8.26 (dt, J = 8.4, 2.0 Hz, 1H), 7.35 (dd, J = 8.3, 4.8 Hz, 1H), 4.03 (d, J = 6.5 Hz, 2H), 2.07–1.94 (m, J = 6.7 Hz, 1H), 0.98 (d, J = 6.6 Hz, 6H). ^13^C NMR (101 MHz, Chloroform-d) δ 178.83, 153.09, 147.59, 145.59, 134.58, 131.71, 123.26, 73.06, 27.75, 18.85.5.

### 4.5. Crystallization Experiments

Co-crystallization experiments were performed in two stages: First, grinding experiments were used for screening, and the resulting products were characterized using infrared spectroscopy. Second, the ground mixtures were dissolved in an appropriate solvent or a mixture of solvents, and then the solvents were allowed to evaporate at room temperature until a crystalline product was formed (see Supplementary Materials). The obtained crystal products were characterized by DSC and SCXRD.

#### 4.5.1. Synthesis of Methyl-N-[(3-pyridinylamino) Thioxomethyl] Carbamate·1,3,5-trifluoro-2,4,6-triiodobenzene co-crystal (**A1·D1**)

Stoichiometric amounts of **A1** (2.5 mg) and **D1** (24 mg) were ground at a 1:4 molar ratio using solvent-assisted grinding (SAG), and the resulting solids were analyzed using IR spectroscopy. The solid mixtures were dissolved in chloroform (3–4 mL) and kept in small vials for slow evaporation at room temperature to obtain single crystals. Crystals suitable for single-crystal X-ray diffraction were obtained after 5–6 days. The melting point of the co-crystal was 168–170 °C.

#### 4.5.2. Synthesis of di-(ethyl-N-[(3-pyridinylamino) Thioxomethyl] Carbamate)·1,3,5-trifluoro-2,4,6-triiodobenzene **(A2)_2_·D1**

Stoichiometric amounts of **A2** (2.5 mg) and **D1** (23 mg) were ground at a 1:4 molar ratio using SAG, and the resulting solids were analyzed using IR spectroscopy. The solid mixtures were dissolved in ethanol (3–4 mL) and kept in small vials for slow evaporation at room temperature to obtain single crystals. Crystals suitable for single-crystal X-ray diffraction were obtained after 5–6 days. The melting point of the co-crystal was 152–154 °C.

#### 4.5.3. Synthesis of Isobutyl-N-[(3-pyridinylamino) Thioxomethyl] Carbamate·1,3,5-trifluoro-2,4,6-triiodobenzene (**A3·D1**)

Stoichiometric amounts of **A3** (2.5 mg) and **D1** (20 mg) were ground using SAG at a 1:4 molar ratio, and the resulting solids were analyzed using IR spectroscopy. The solid mixtures were dissolved in ethanol (3–4 mL) and kept in small vials for slow evaporation at room temperature to obtain single crystals. Crystals suitable for single-crystal X-ray diffraction were obtained after 3 weeks. The melting point of the co-crystal was 171–174 °C.

#### 4.5.4. Synthesis of di-(methyl-N-[(3-pyridinylamino) Thioxomethyl] Carbamate)·1,2,4,5-tetrafluoro-3,6-diiodobenzene Chloroform Solvate **(A1)_2_·D2**

Stoichiometric amounts of **A1** (2.5 mg) and **D2** (8 mg) were ground at a 1:2 molar ratio using SAG, and the resulting solids were analyzed using IR spectroscopy. The solid mixtures were dissolved in chloroform (3–4 mL) and kept in small vials for slow evaporation at room temperature to obtain single crystals. Crystals suitable for single-crystal X-ray diffraction were obtained after 10 days. The melting point of the co-crystal was 158–160 °C.

#### 4.5.5. Synthesis of Ethyl-N-[(3-pyridinylamino) Thioxomethyl] Carbamate·1,2,4,5-tetrafluoro-3,6-diiodobenzene **A2·D2**

Stoichiometric amounts of **A2** (2.5 mg) and **D2** (9 mg) were ground at a 1:2 molar ratio using SAG, and the resulting solids were analyzed using IR spectroscopy. The solid mixtures were dissolved in chloroform (3–4 mL) and kept in small vials for slow evaporation at room temperature to obtain single crystals. Crystals suitable for single-crystal X-ray diffraction were obtained after 1 week. The melting point of the co-crystal was 106–108 °C.

#### 4.5.6. Synthesis of di-(isobutyl-N-[(3-pyridinylamino) Thioxomethyl] Carbamate)·1,2,4,5-tetrafluoro-3,6-diiodobenzene **(A3)_2_·D2**

Stoichiometric amounts of **A3** (2.5 mg) and **D2** (8 mg) were ground using SAG at a 1:2 molar ratio, and the resulting solids were analyzed using IR spectroscopy. The solid mixture was dissolved in chloroform (3–4 mL) and kept in small vials for slow evaporation at room temperature to obtain single crystals. Crystals suitable for single-crystal X-ray diffraction were obtained after 1 week. The melting point of the co-crystal was 106**–**108 °C.

## 5. Conclusions

We investigated a series of three parent molecules, as well as five pure co-crystals and one co-crystal solvate, from R-*N*-[(3-pyridinylamino) thioxomethyl] carbamates with fluoroiodobenzene co-formers. Analysis of the parent molecules (**A1**–**A3**) showed that increasing the alkyl chain length does not significantly impact the overall supramolecular aggregation of the molecular building blocks. Overall, the N–H⋯Npyr hydrogen bond was the dominant intermolecular interaction, as it appeared in all three structures, producing dimeric units in each case.

Co-crystallization experiments showed that the N-H⋯Npyr interaction was present in 3/6 co-crystals with additional halogen-bond interactions. In cases where the parent molecules did not form N–H⋯Npyr chains, hydrogen-bonded dimers through N-H···S=C and N-H···O=C stabilized the architectures. Co-crystals with **D1** displayed varying halogen-bonding patterns, i.e., (I⋯S=C, I⋯O=C), (I⋯Npyr), and (I⋯Npyr, I⋯C=S) for **A1·D1**, **(A2)_2_·D1**, and **A3·D1**, respectively. Co-crystallization with **D2** in **(A1)_2_·D2** and **A2·D2** displayed binding preference to C=S, which is the second-best acceptor, forming an I⋯C=S halogen bond, while the structure **(A3)_2_·D2** shows binding preference to the best acceptor Npyr. I⋯Npyr was found in 3/6 structures, I⋯C=S in 4/6 structures, and I⋯O=C only in 1 case. It is worth noting that the I⋯C=S halogen bond coexisted twice with I⋯Npyr and I⋯O=C, separately. CSD contact search revealed that the I⋯Npyr was most prevalent, followed by I⋯S=C, and then I⋯O=C; this trend is similar to what we observed for our structures.

Calculated molecular electrostatic potentials and % van der Waals radii suggest the formation of halogen bonding to the acceptors in the order Npyr > C=S > C=O. Overall, to a greater extent, the co-crystals exhibited I⋯C=S halogen bond interactions even when the MEP calculations favored the Npyr acceptor by ∼60 kJ/mol, suggesting that it is crucial to consider additional factors that affect co-crystallization outcomes, such as geometry. Despite not establishing a consistent synthon prediction trend for our system, we established that halogen-bond donors are competitive for binding sites even in the presence of strong hydrogen-bond donors, but synthon preference in the presence of multiple acceptors is not as straightforward as is the case for prediction in simpler systems.

## Figures and Tables

**Table 1:** XA distances and angles from crystal structures.

Co-Crystal	Halogen Bond	Experimental XA Distance		XA Angle
		(Å)	% vdW Reduction	(°)
**A1·D1**	I···S=C	3.310(3)	12.2	161.9(3)
	I···O=C	3.168(9)	9.1	154.2(4)
**(A2)_2_·D1**	I···Npyr	2.877(2)	18.2	177.2(8)
**A3·D1**	I···S=C	3.363(6)	10.9	171.9(7)
	I···Npyr	2.927(3)	16	176.2(1)
**(A1)_2_·D2**	I···S=C	3.222(1)	14.6	169.2(1)
**A2·D2**	I···S=C	3.248(7)	13.8	170.0(6)
**(A3)_2_·D2**	I···Npyr	2.802(6)	20.5	177.8(2)

## Data Availability

Crystallographic data (CCDC 2154746, 2154747, 2154748, 2154749, 2154750, 2154751, 2154752, 2154753) can be obtained free of charge via www.ccdc.cam.ac.uk/data_request/cif (accessed on 24 February 2021), by emailing data_request@ccdc.cam.ac.uk, or by contacting The Cambridge Crystallographic Data Centre, 12 Union Road, Cambridge CB2 1EZ, UK; fax: +44-1223-336033.

## Supplementary Materials

The following are available online at https://www.mdpi.com/article/10.3390/molecules27123685/s1. Figures S1–S3: 1H and 13C NMR spectra of **A1**, **A2**, and **A3**, respectively. Tables S1 and S2: Crystallographic data. Table S3: Hydrogen- and halogen-bond parameters of the eight structures. Figure S4: Schematic of the contact descriptors used for the CSD search. Figures S5 and S6: Histograms showing bond length against frequency, plotted using Mercury.

## Sample Availability

Samples of the compounds are not available from the authors.

## References

[1] Ardila-Fierro K.J., André V., Tan D., Duarte M.T., Lancaster R.W., Karamertzanis P.G., Friščić T. (2015). Molecular Recognition of Steroid Hormones in the Solid State: Stark Differences in Cocrystallization of β-Estradiol and Estrone. Cryst. Growth Des..

[2] Pramanik T., Sarkar S., Guru Row T.N. (2022). Halogen Bonded Network Modulating the Mechanical Property Elastic and Plastic Bending in Nonconventional Molecular Solid Solutions. Cryst. Growth Des..

[3] Reddy C.M., Padmanabhan K.A., Desiraju G.R. (2006). Structure−Property Correlations in Bending and Brittle Organic Crystals. Cryst. Growth Des..

[4] Saha S., Desiraju G.R. (2017). σ-Hole and π-Hole Synthon Mimicry in Third-Generation Crystal Engineering: Design of Elastic Crystals. Chem.—Eur. J..

[5] Yadava K., Qin X., Liu X., Vittal J.J. (2019). Straight, bendable and bent organic crystals. Chem. Commun..

[6] Ghosh S., Mishra M.K., Kadambi S.B., Ramamurty U., Desiraju G.R. (2015). Designing Elastic Organic Crystals: Highly Flexible Polyhalogenated N-Benzylideneanilines. Angew. Chem. Int. Ed..

[7] Juneja N., Shapiro N.M., Unruh D.K., Bosch E., Groeneman R.H., Hutchins K.M. (2022). Controlling Thermal Expansion in Supramolecular Halogen-Bonded Mixed Cocrystals through Synthetic Feed and Dynamic Motion. Angew. Chem. Int. Ed..

[8] Liu Y., Ward M.D. (2009). Molecular Capsules in Modular Frameworks. Cryst. Growth Des..

[9] Aakeröy C.B., Beatty A.M., Helfrich B.A., Nieuwenhuyzen M. (2003). Do Polymorphic Compounds Make Good Cocrystallizing Agents? A Structural Case Study that Demonstrates the Importance of Synthon Flexibility. Cryst. Growth Des..

[10] Sreekanth B.R., Vishweshwar P., Vyas K. (2007). Supramolecular synthon polymorphism in 2:1 co-crystal of 4-hydroxybenzoic acid and 2,3,5,6-tetramethylpyrazine. Chem. Commun..

[11] Mukherjee A., Desiraju G.R. (2011). Synthon polymorphism and pseudopolymorphism in co-crystals. The 4,4′-bipyridine–4-hydroxybenzoic acid structural landscape. Chem. Commun..

[12] Burrows J.A., Pauling L. (1941). The nature of the chemical bond and the structure of molecules and crystals. Nature.

[13] Aakeröy C.B., Spartz C.L., Dembowski S., Dwyre S., Desper J. (2015). A systematic structural study of halogen bonding versus hydrogen bonding within competitive supramolecular systems. IUCrJ.

[14] Galek P.T.A., Allen F.H., Fábián L., Feeder N. (2009). Knowledge-based H-bond prediction to aid experimental polymorph screening. CrystEngComm.

[15] Hunter C.A. (2004). Quantifying Intermolecular Interactions: Guidelines for the Molecular Recognition Toolbox. Angew. Chem. Int. Ed..

[16] Musumeci D., Hunter C.A., Prohens R., Scuderi S., McCabe J.F. (2011). Virtual cocrystal screening. Chem. Sci..

[17] DeHaven B.A., Chen A.L., Shimizu E.A., Salpage S.R., Smith M.D., Shimizu L.S. (2019). Interplay between Hydrogen and Halogen Bonding in Cocrystals of Dipyridinylmethyl Oxalamides and Tetrafluorodiiodobenzenes. Cryst. Growth Des..

[18] Martí-Rujas J., Colombo L., Lü J., Dey A., Terraneo G., Metrangolo P., Pilati T., Resnati G. (2012). Hydrogen and halogen bonding drive the orthogonal self-assembly of an organic framework possessing 2D channels. Chem. Commun..

[19] Aakeröy C.B., Wijethunga T.K., Haj M.A., Desper J., Moore C. (2014). The structural landscape of heteroaryl-2-imidazoles: Competing halogen- and hydrogen-bond interactions. CrystEngComm.

[20] Gunawardana C.A., Desper J., Sinha A.S., Ðaković M., Aakeröy C.B. (2017). Competition and selectivity in supramolecular synthesis: Structural landscape around 1-(pyridylmethyl)-2,2′-biimidazoles. Faraday Discuss..

[21] Abeysekera A.M., Averkiev B.B., Sinha A.S., Le Magueres P., Aakeröy C.B. (2021). Establishing Halogen-Bond Preferences in Molecules with Multiple Acceptor Sites. ChemPlusChem.

[22] De Silva V., Averkiev B.B., Sinha A.S., Aakeröy C.B. (2021). The Balance between Hydrogen Bonds, Halogen Bonds, and Chalcogen Bonds in the Crystal Structures of a Series of 1,3,4-Chalcogenadiazoles. Molecules.

[23] Stanley C.W., Thornton J.S., Katague D.B. (1972). Gas-chromatographic method for residues of Baygon and metabolites in plant tissues. J. Agric. Food Chem..

[24] Teixeira M.A., Barrault L., Rodríguez O., Carvalho C.C., Rodrigues A.E. (2014). Perfumery Radar 2.0: A Step toward Fragrance Design and Classification. Ind. Eng. Chem. Res..

[25] Axthammer Q.J., Krumm B., Klapötke T.M. (2015). Synthesis of Energetic Nitrocarbamates from Polynitro Alcohols and Their Potential as High Energetic Oxidizers. J. Org. Chem..

[26] Popović M., Nierkens S., Pieters R., Uetrecht J. (2004). Investigating the Role of 2-Phenylpropenal in Felbamate-Induced Idiosyncratic Drug Reactions. Chem. Res. Toxicol..

[27] Parker R.J., Hartman N.R., Roecklein B.A., Mortko H., Kupferberg H.J., Stables J., Strong J.M. (2005). Stability and Comparative Metabolism of Selected Felbamate Metabolites and Postulated Fluorofelbamate Metabolites by Postmitochondrial Suspensions. Chem. Res. Toxicol..

[28] Tegeler T., El Rassi Z. (2001). On-Column Trace Enrichment by Sequential Frontal and Elution Electrochromatography. 1. Application to Carbamate Insecticides. Anal. Chem..

[29] Aly O.M., El-Dib M.A. (1972). Studies of The Persistence of Some Carbamate Insecticides in The Aquatic Environment. Fate of Organic Pesticides in the Aquatic Environment.

[30] Shamim M., Meléndez J., Sappington K., Ruhman M. (2014). Conducting Ecological Risk Assessments of Urban Pesticide Uses. Describing the Behavior and Effects of Pesticides in Urban and Agricultural Settings.

[31] Zhang J.J., Yang H. (2021). Advance in Methodology and Strategies To Unveil Metabolic Mechanisms of Pesticide Residues in Food Crops. J. Agric. Food Chem..

[32] Ghosh A.K., Brindisi M. (2015). Organic Carbamates in Drug Design and Medicinal Chemistry. J. Med. Chem..

[33] Matošević A., Bosak A. (2020). Carbamate group as structural motif in drugs: A review of carbamate derivatives used as therapeutic agents. Arch. Ind. Hyg. Toxicol..

[34] Bennion J.C., Matzger A.J. (2021). Development and Evolution of Energetic Cocrystals. Acc. Chem. Res..

[35] Axthammer Q.J., Klapötke T.M., Krumm B., Reith T. (2016). Energetic Sila-Nitrocarbamates: Silicon Analogues of Neo-Pentane Derivatives. Inorg. Chem..

[36] Klapötke T.M., Krumm B., Unger C.C. (2019). Energetic Metal and Nitrogen-Rich Salts of the Pentaerythritol Tetranitrate Analogue Pentaerythritol Tetranitrocarbamate. Inorg. Chem..

[37] Aitipamula S., Banerjee R., Bansal A.K., Biradha K., Cheney M.L., Choudhury A.R., Desiraju G.R., Dikundwar A.G., Dubey R., Duggirala N. (2012). Polymorphs, Salts, and Cocrystals: What’s in a Name?. Cryst. Growth Des..

[38] Aakeröy C.B., Fasulo M.E., Desper J. (2007). Cocrystal or Salt: Does It Really Matter?. Mol. Pharm..

[39] Desiraju G.R. (2013). Crystal Engineering: From Molecule to Crystal. J. Am. Chem. Soc..

[40] Zhang Y.-M., Yang L.-Z., Lin Q., Wei T.-B., Wang D.-Q. (2006). Synthesis and the crystal structure of N-ethoxycarbonyl-N′-(3-pyridyl)thiourea. Youji Huaxue.

[41] Spek A. (2009). Structure validation in chemical crystallography. Acta Crystallogr. Sect. D.

[42] Bondi A. (1964). van der Waals Volumes and Radii. J. Phys. Chem..

[43] Groom C.R., Bruno I.J., Lightfoot M.P., Ward S.C. (2016). The Cambridge Structural Database. Acta Crystallogr. Sect. B.

[44] Macrae C.F., Sovago I., Cottrell S.J., Galek P.T.A., McCabe P., Pidcock E., Platings M., Shields G.P., Stevens J.S., Towler M. (2020). Mercury 4.0: From visualization to analysis, design and prediction. J. Appl. Crystallogr..

[45] Wavefunction, Inc. Irvine, CA 92612, USA. https://www.wavefun.com/products/spartan.html.

[46] Wei T.B., Lin Q., Zhang Y.M., Wang H. (2004). Efficient and Novel Synthesis of *N*-Aryl-*N*′-ethoxycarbonylthiourea and Arene-bis-ethoxycarbonylthiourea Derivatives Catalyzed by TMEDA. Synth. Commun..

[47] Ren Y.-H., Song J.-R., Xu K.-Z., Ma H.-X., Huang J., Fu D.-W., Hu H.-M. (2007). Preparation, Crystal Structure and Theoretical Calculation of *N*-(Pyrimidin-2-yl)-N′-methoxycarbonyl-thiourea. Chin. J. Chem..

