# Supplementary material for: Influence of Multiple Binding Sites on the Supramolecular Assembly of N-[(3-pyridinylamino) Thioxomethyl] Carbamates
Source: Molecules. 2022 Jun 8;27(12):3685. doi: 10.3390/molecules27123685 (PMC9228572; doi:10.3390/molecules27123685)
Supplement: Supplementary file 1 [file molecules-27-03685-s001.zip › paper1_Supplementary Information_molecules_Kelly_draft_4_final.pdf]

molecules

MDPI

Supplementary Information

Influence of multiple binding sites on the supramolecular assembly of *N*-[(3-pyridinylamino) thioxomethyl] carbamates

Kelly N. Shunje, Boris B. Averkiev, and Christer B. Aakeröy *

Department of Chemistry, Kansas State University, Manhattan, KS 66506, USA; knshunje@ksu.edu (K.N.S); averkiev@ksu.edu (B.B.A)
* Correspondence: aakeroy@ksu.edu (C.B.A); Tel.: +1-785-532-6096

S1 General Remarks
S2 Spectra Data
S3 Crystallographic information
S4 CSD analysis

1. General Remarks

Commercial reagents were purchased as reagent–grade and used without further purification. All solvents utilized in this study were purchased commercially as technical grade and used as is without further purification. Targets were synthesized by modified versions of previously reported synthetic routes as described when referenced. Melting points were measured using a TA Instruments DSC Q20 differential scanning calorimeter. Nuclear magnetic resonance (NMR) data were collected using either a Bruker Ascend 400 MHz or Varian Unity Plus 400 MHz spectrometer. The residual solvent peak was used as the internal reference for 1H and 13C NMR (CDCl3: δH=7.26ppm, δC=77.16ppm). Single crystal X-ray diffraction data were collected using a Rigaku XtaLAB Synergy-S1 diffractometer. Data collection parameters are outlined in crystallographic information table. The structure was solved using Olex23 with the SHELXT4 structure solution program using Intrinsic Phasing and refined with the SHELXL5 refinement package using Least Squares minimization.

2. Spectral Data

2.1. Synthesis of methyl-*N*-[(3-pyridinylamino) thioxomethyl] carbamate (A1)

^{1}H NMR (400 MHz, Chloroform-d) δ 11.50 (s, 1H), 8.70 (d, J = 2.6 Hz, 1H), 8.51 (dd, J = 4.9, 1.5 Hz, 1H), 8.39 (s, 1H), 8.25 (ddd, J = 8.3, 2.8, 1.5 Hz, 1H), 7.36 (dd, J = 8.3, 4.8 Hz, 1H), 3.88 (s, 3H)

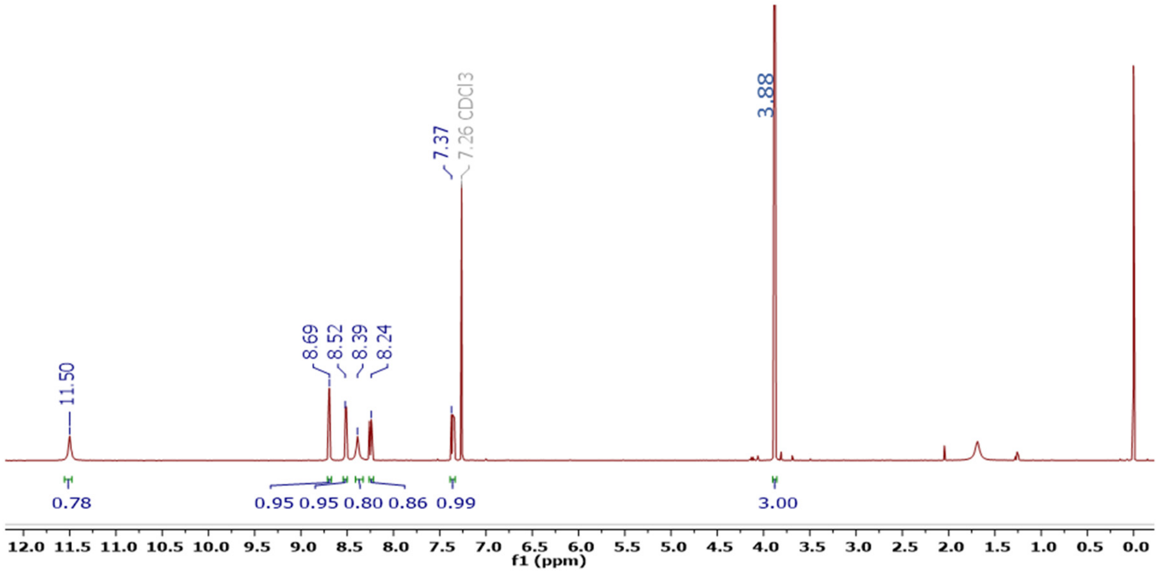

^{13}C NMR (101 MHz, CDCl3) δ 178.57, 153.31, 147.68, 145.60, 134.52, 131.73, 123.29, 53.74.

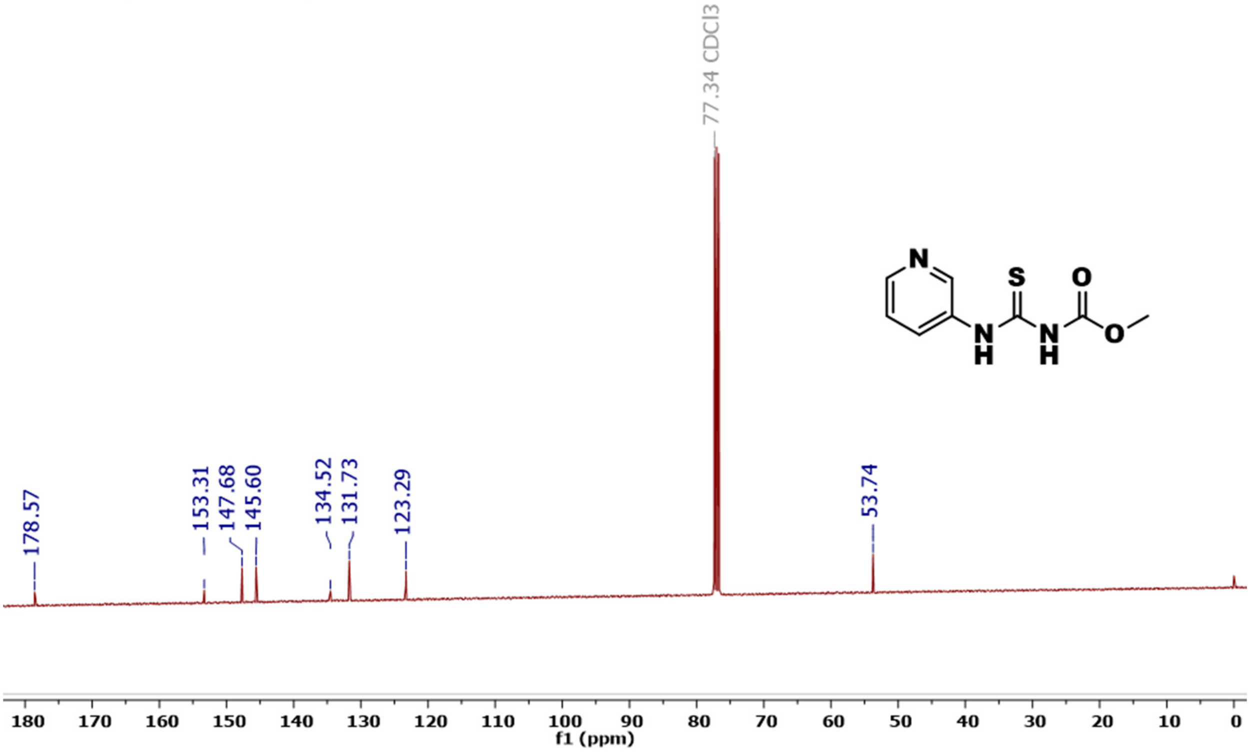

*Figure S1 H-NMR (top) and C13-NMR (bottom) of methyl-*N*-[(3-pyridinylamino) thioxomethyl] carbamate (A1)*

2.2. Synthesis of ethyl-*N*-[(3-pyridinylamino) thioxomethyl] carbamate (A2)

^{1}H NMR (400 MHz, Chloroform-*d*) δ 11.56 (s, 1H), 8.71 (d, *J* = 2.6 Hz, 1H), 8.60 – 8.56 (m, 1H), 8.52 (dd, *J* = 4.8, 1.5 Hz, 1H), 8.26 (ddd, *J* = 8.4, 2.8, 1.6 Hz, 1H), 7.39 – 7.31 (m, 1H), 4.31 (q, *J* = 7.1 Hz, 2H), 1.37 (t, *J* = 7.1 Hz, 3H).

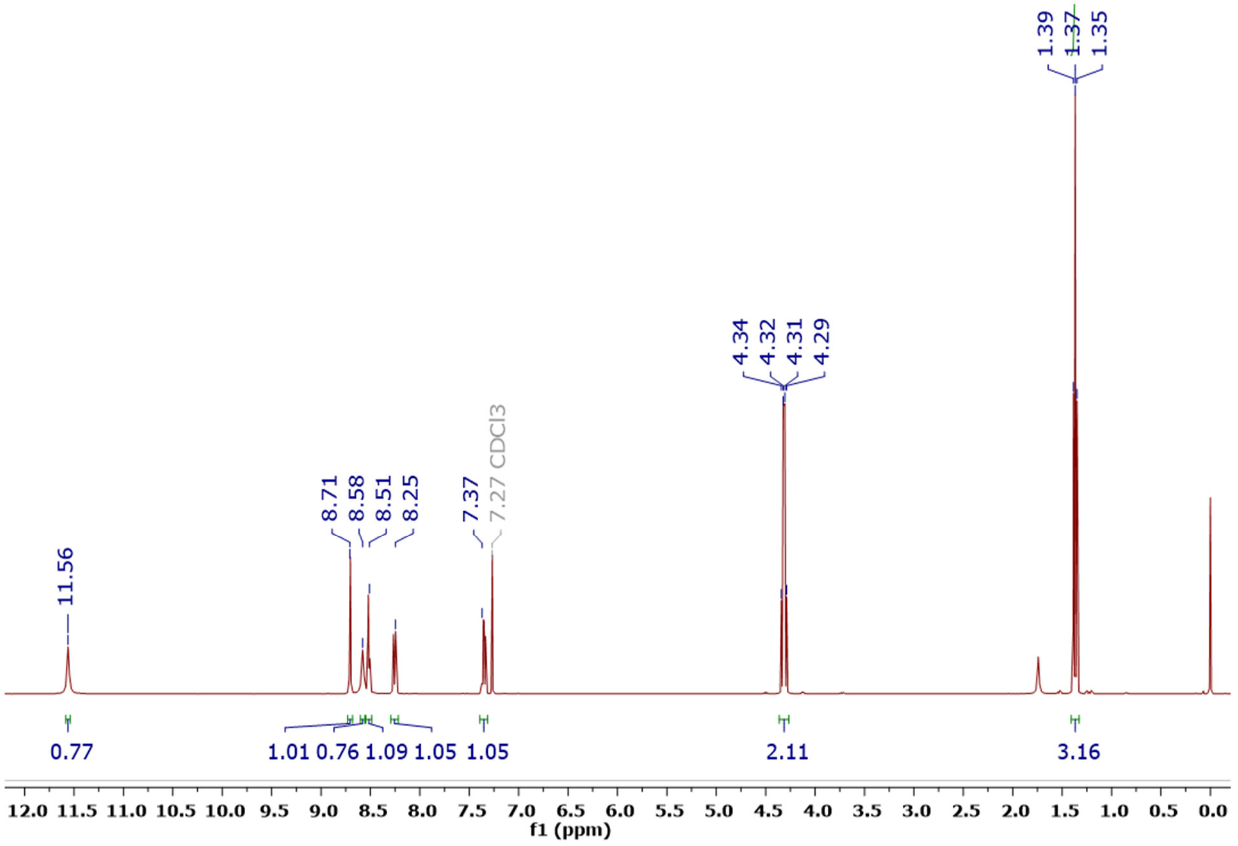

^{13}C NMR (101 MHz, $CDCl_3$) δ 178.81, 152.98, 147.59, 145.60, 134.59, 131.72, 123.26, 63.31, 14.21

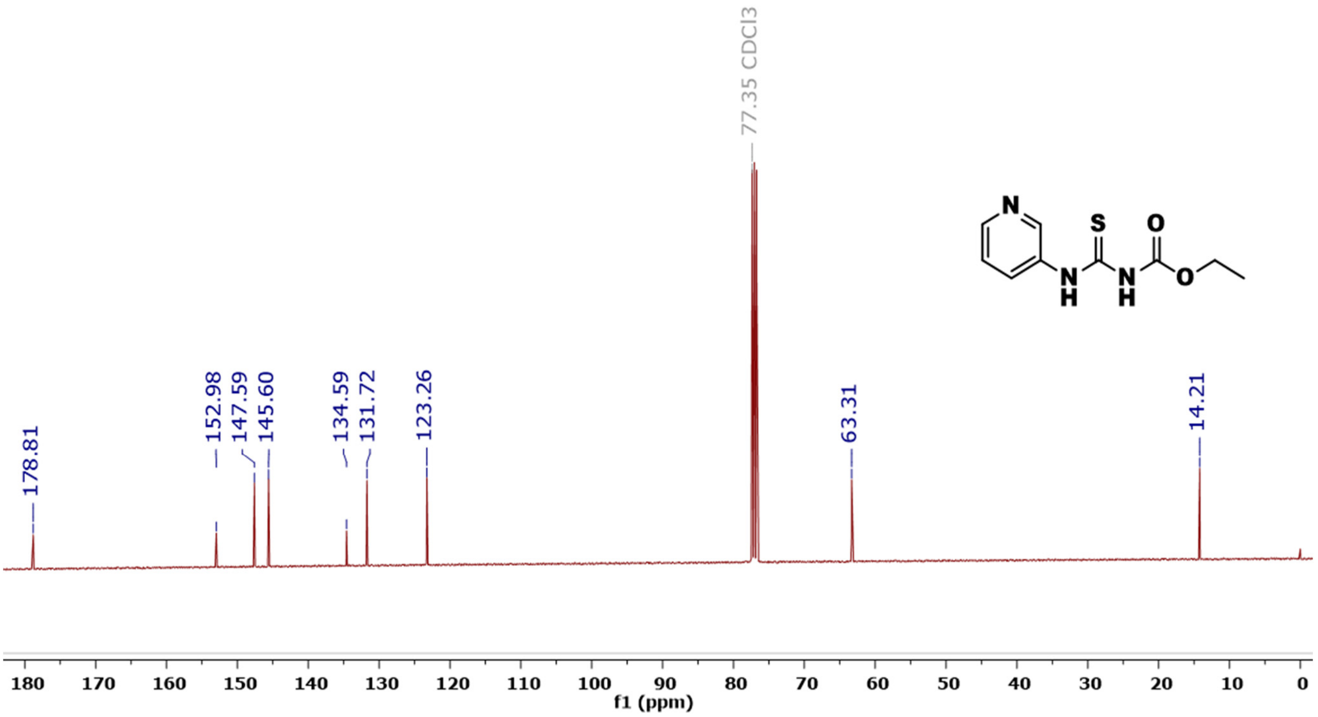

*Figure S2 H-NMR (top) and C13-NMR (bottom) of ethyl-*N*-[(3-pyridinylamino) thioxomethyl] carbamate (A2)*

2.3. Synthesis of isobutyl-*N*-[(3-pyridinylamino) thioxomethyl] carbamate (A3)

^{1}H NMR (400 MHz, Chloroform-*d*) δ 11.56 (s, 1H), 8.71 (d, *J* = 2.6 Hz, 1H), 8.65 (s, 1H), 8.51 (d, *J* = 4.8 Hz, 1H), 8.26 (dt, *J* = 8.4, 2.0 Hz, 1H), 7.35 (dd, *J* = 8.3, 4.8 Hz, 1H), 4.03 (d, *J* = 6.5 Hz, 2H), 2.07 – 1.94 (m, *J* = 6.7 Hz, 1H), 0.98 (d, *J* = 6.6 Hz, 6H).

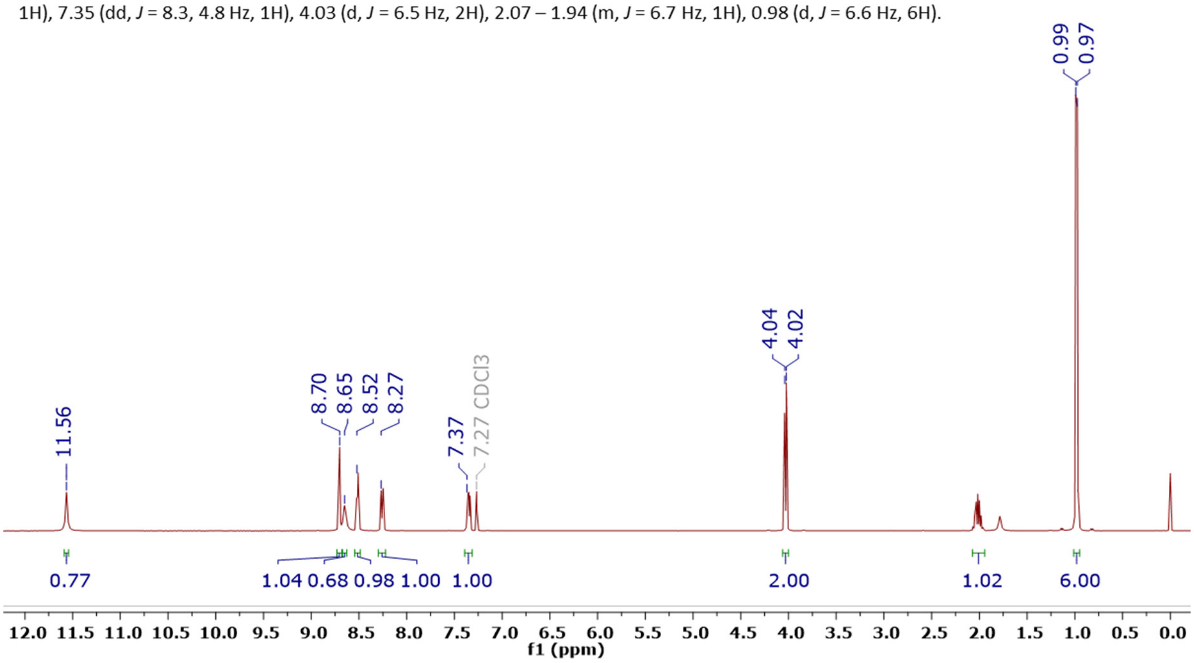

^{13}C NMR (101 MHz, Chloroform-*d*) δ 178.83, 153.09, 147.59, 145.59, 134.58, 131.71, 123.26, 73.06, 27.75, 18.85.

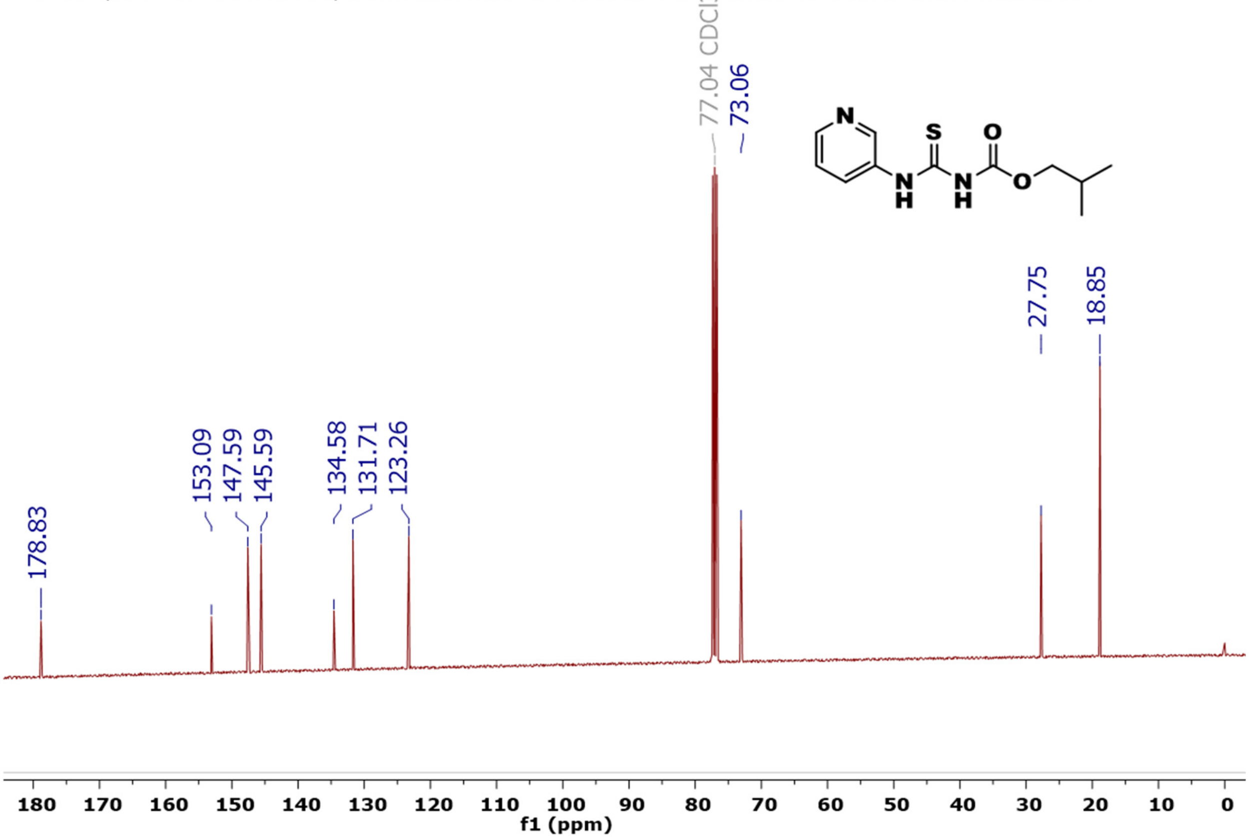

*Figure S3 H-NMR (top) and C13-NMR (bottom) of isobutyl-*N*-[(3-pyridinylamino) thioxomethyl] carbamate (A3)*

3. Crystallographic information

Table S1 Crystallographic information of targets explored in this study

Compounds	A1	A3
Identification code	exp_231	Exp_334
Molecular formula	$C_8H_9N_3O_2S$	$C_{11}H_{15}N_3O_2S$
Formula weight (g mol^{-1})	211.24	253.32
Crystal system	orthorhombic	triclinic
Space group (No.)	*Fdd2*	*P- 1* (No. 2)
a (Å)	28.61372(17)	9.5250(3)
b (Å)	19.21403(12)	10.2023(2)
c (Å)	6.92761(4)	14.3104(2)
α (°)	90	73.7596(18)
β (°)	90	78.554(2)
γ (°)	90	78.444(2)
V (Å^3)	3808.70(4)	1293.01(6)
Z	16	4
ρ_{calc} (g cm^{-3})	1.474	1.301
μ (Cu Kα) (mm^{-1})	2.866	2.195
F (000)	1760.0	536.0
Crystal size (mm)	0.328 × 0.231 × 0.111	0.150 x 0.050 x 0.020
Temperature (K)	200 (10)	140 (2)
Radiation (Å)	CuKα, 1.54184	CuKα, 1.54174
Theta range for data collection	5.547 to 77.019°	3.254 to 77.577°
Index ranges	-35 ≤ h ≤ 36, -23 ≤ k ≤ 23, -8 ≤ l ≤ 8	-11<=h<=12, -12<=k<=12, -18<=l<=16
Final R indices [I>2.0 σ(I)]	R_1=0.0225, wR_2=0.0645	R_1=0.0524, wR_2=0.1537
Final R indices [all data]	R_1=0.0225, wR_2=0.0645	R_1=0.0577, wR_2= 0.1577
Tot ref., uniq. ref, R (int)	16723, 1972, 0.0181	22321, 5358,0.0438
Max. and min. transmission	1.000 and 0.265	1.00000 and 0.74851
Data/restraints/parameters	1972/1/137	5358 / 1 / 322
Goodness-of-fit on F^2	1.072	1.038
Largest diff. peak/hole (e. Å^{-3})	0.192/-0.246	0.688/-0.717

Table S2 Crystallographic information of cocrystals obtained in this study

**Compounds**	**A1·D1**	**$(A2)_2$·D1**	**A3·D1**	**$(A1)_2$·D2**	**A2·D2**	**$(A3)_2$·D2**
**Identification code**	exp_335c	exp_248	exp_308	exp_363	exp_351	Exp_393
**Empirical formula**	$C_{14}F_3 I_3 H_9 N_3 O_2 S$	$C_{12}H_{11}F_{1.5}I_{1.5}N_3O_2S$	$C_{17}F_3 I_3 H_{15}N_3 O_2 S$	$C_{12}F_2 ClI H_{10}N_3 O_2 S$	$C_{15}F_4 I_2 H_{11}N_3 O_2 S$	$C_{14}F_2 IH_{15}N_3 O_2 S$
**Formula weight ($g\ mol^{-1}$)**	721.00	480.15	763.08	531.54	627.13	454.25
**Crystal system**	monoclinic	orthorhombic	triclinic	monoclinic	monoclinic	monoclinic
**Space group (No.)**	*$P2_1/c$*	*Pnna*	*P- 1* (No. 2)	*$P2_1/c$*	*$P2_1/c$*	*$P2_1/c$*
**a (Å)**	4.3912(3)	17.4398(3)	9.19100(10)	4.20680(10)	4.40696(4)	19.6991(9)
**b (Å)**	14.6099(10)	35.2133(6)	11.04700(10)	26.0337(5)	27.4127(3)	4.78474(13)
**c (Å)**	15.3389(9)	5.09340(10)	12.41600(10)	15.3735(3)	15.48356(14)	20.6316(10)
**α (°)**	90	90	64.5040(10)	90	90	90
**β (°)**	96.621(6)	90	88.3510(10)	94.498	91.4598(9)	116.983(6)
**γ (°)**	90	90	79.9240(10)	90	90	90
**V ($Å^3$)**	977.50(11)	3127.92(10)	1118.78(2)	1678.50(6)	1869.91(3)	1732.94(15)
**Z**	2	8	2	4	4	4
**ρ_{calc} ($g\ cm^{-3}$)**	2.450	2.039	2.265	2.103	2.228	1.741
**μ (Cu Kα) (mm^{-1})**	4.945	25.315	34.185	20.875	27.990	15.941
**F (000)**	664	1832.0	712	1028	1184	892
**Crystal size (mm)**	0.16 x 0.01 x 0.01	0.155 × 0.06 × 0.035	0.159 x 0.108 x 0.059	0.121 x 0.013 x 0.01	0.098 x 0.052 x 0.033	0.255 x 0.031 x 0.022
**Temperature (K)**	140.00(10)	170.00(10)	130.00(10)	139.7(6)	140.00(10)	200.00(10)
**Radiation (Å)**	CuKα, 1.54184	CuKα, 1.54184	CuKα, 1.54184	CuKα, 1.54184	CuKα, 1.54184	CuKα, 1.54184
**Theta range for data collection/°**	2.674 to 30.814	2.510 to 77.122	3.950 to 77.463	4.457 to 77.733	3.224 to 77.545	2.517 to 77.810
**Index ranges**	-5<=h<=5, -20<=k<=20, -19<=l<=21	-21 ≤ h ≤ 16, -44 ≤ k≤ 39, -6 ≤ l ≤ 6	-11<=h<=11, -13<=k<=13, -15<=l<=15	-5<=h<=3, -32<=k<=32, -19<=l<=19	-5<=h<=5, -33<=k<=34, -18<=l<=19	-24<=h<=24, -6<=k<=4, -26<=l<=25
**Final R indices [I>2.0 σ(I)]**	R_1=0.0439, wR_2=0.0439	R_1 = 0.0213 wR_2 = 0.0598	R_1=0.0237 wR_2= 0.0648	R_1=0.0403 wR_2= 0.1071	R_1=0.0198 wR_2= 0.0511	R_1=0.0517 wR_2= 0.1312
**Final R indices [all data]**	R_1=0.0514 wR_2=0.1130	R_1 = 0.0220 wR_2 = 0.0603	R_1= 0.0239 wR_2= 0.0650	R_1=0.0417 wR_2= 0.1082	R_1=0.0203 wR_2= 0.0515	R_1=0.0575 wR_2= 0.1344
**Tot ref., uniq. ref, R (int)**	15121, 4704, 0.0596	17973, 3307,0.0256	42499, 4731, 0.0493	26685, 3561, 0.0459	22839, 4000, 0.47941	18918, 3609, 0.0625
**Max. and min. transmission**	1.00000 and 0.60523	1.00000 and 0.50257	1.00000 and 0.22072	0.920 and 0.327	1.00000 and 0.47941	1.000 and 0.408
**Data/restraints/ parameters**	4704 / 2 / 236	3307/0/193	4731 / 0 / 265	3561/142/262	4000 / 0 / 246	3609 / 0 / 210
**Goodness-of-fit**	1.006	1.062	1.050	1.100	1.047	1.118
**Largest diff. peak/hole (e. $Å^{-3}$)**	1.309/-1.013	0.664/-1.096	1.165/-1.053	1.206/-0.929	0.933/-0.945	1.210/-1.471

Table S3 Hydrogen and halogen bond parameters of the eight crystal structures

	DH/X---A	D/X/Ch---A (Å)	<[DH/X/Ch---A] (°)
**A1**	C(3)-H(3)···S(9)	3.821(2)	153.0
	C(4)-H(4)···O(12)	3.436(2)	172.3
	N(7)-H(7)···O(12)	2.674(2)	140.0
	N(10)-H(10)···N(2)	2.891(2)	174.0
**A3**	C(4A)-H(4A)···S(9A)	3.666(2)	131.9
	N(7)-H(7)···O(12)	2.633(3)	140.9
	N(10)-H(10)···N(2A)	2.899(3)	172.5
**A1·D1**	C(3)-H(3)···S(9)	3.623(1)	134.7
	N(7)-H(7)···O(12)	2.664(1)	134.4
	N(10)-H(10)···N(2)	2.877(1)	171.6
	I(16)···S(9)	3.310(3)	
	C(15)-I(16)···S(9)		161.9(3)
	I(24)···O(12)	3.168(9)	
	C(24)-I(24)···O(12)		154.2(4)
**(A2)$_2$·D1**	C(1)-H(1)···S(9)	3.695(3)	159.2
	N(7)-H(7)···O(12)	2.713(3)	132.9
	N(7)-H(7)···O(12)	3.095(3)	131.2
	N(10)-H(10)···S(9)	3.311(2)	169.3
	I(21)···N(2)	2.877(2)	
	C(20)-I(21)···N(2)		177.2(8)
**A3·D1**	C(4)-H(4)···S(9)	3.774(3)	151.5
	C(5)-H(5)···F(25)	3.396(3)	165.4
	N(7)-H(7)···N(2)	3.282(3)	128.1
	N(7)-H(7)···O(12)	2.670(3)	134.2
	N(10)-H(10)···S(9)	3.366(2)	160.9
	I(27)···S(9)	3.363(6)	
	C(26)-I(27)···S(9)		171.9(7)
	I(19)···N(2)	2.927(3)	
	C(18)-I(19)···N(2)		176.2(1)
**(A1)$_2$·D2**	N(7)-H(7)···O(12)	2.653(5)	136.4
	N(10)-H(10)···N(2)	2.880(5)	173.9
	I(16)···S(9)	3.222(1)	
	C(15)-I(16)···S(9)		169.2(1)
**A2·D2**	N(7)-H(7)···O(12)	2.639(3)	135.9
	N(10)-H(10)···N(2)	2.889(3)	167.3
	I(17)···S(9)	3.248(7)	
	C(16)-I(17)···S(9)		170.0(6)
**(A3)$_2$·D2**	C(5)-H(5)···S(9)	3.199(6)	126.2
	N(7)-H(7)···O(12)	2.680(7)	141.6
	N(10)-H(10)···S(9)	3.320(5)	169.3
	I(19)···N(2)	2.802(6)	
	C(18)-I(19)···N(2)		177.8(2)

4. CSD analysis of I···Npyr, I···S=C, and I···O=C bond lengths

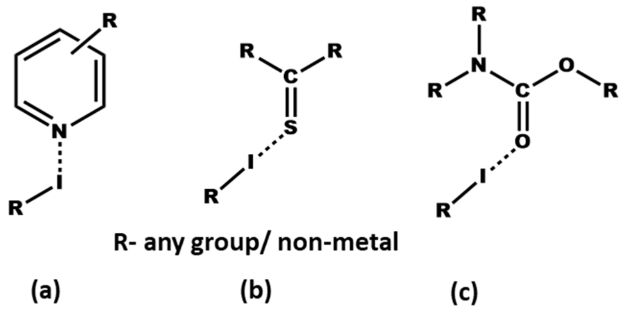

Figure S4 Contact descriptor used for CSD search (a) I···Npyr contact, (b) I···S=C contact, and (c) I···O=C contact

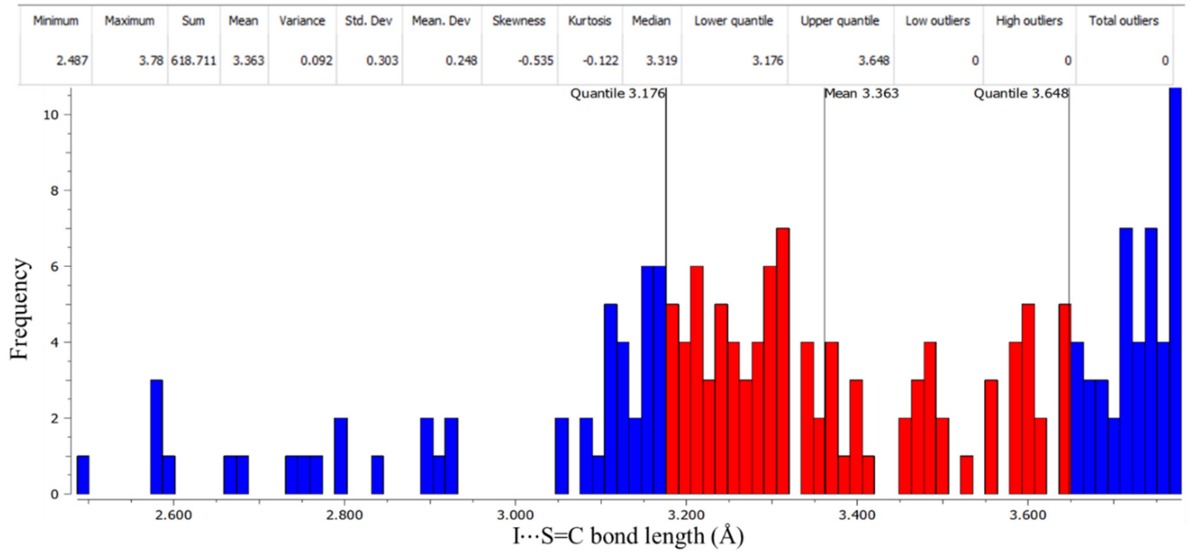

Minimum	Maximum	Sum	Mean	Variance	Std. Dev	Mean. Dev	Skewness	Kurtosis	Median	Lower quantile	Upper quantile	Low outliers	High outliers	Total outliers
2.487	3.78	618.711	3.363	0.092	0.303	0.248	-0.535	-0.122	3.319	3.176	3.648	0	0	0

Figure S5 Histogram showing bond length against frequency for I···S=C halogen bond

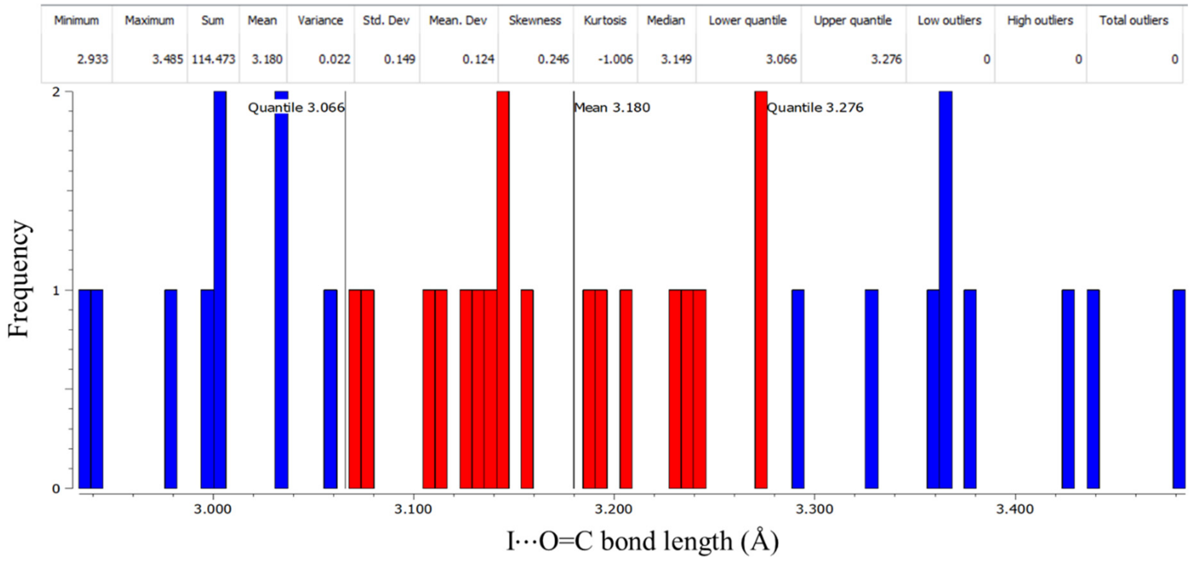

Minimum	Maximum	Sum	Mean	Variance	Std. Dev	Mean. Dev	Skewness	Kurtosis	Median	Lower quantile	Upper quantile	Low outliers	High outliers	Total outliers
2.933	3.485	114.473	3.180	0.022	0.149	0.124	0.246	-1.006	3.149	3.066	3.276	0	0	0

Figure S6 Histogram showing bond length against frequency for I···O=C halogen bond
